# Reflections on a Community Engagement Strategy for Mass Antimalarial Drug Administration in Cambodia

**DOI:** 10.4269/ajtmh.17-0428

**Published:** 2017-11-20

**Authors:** Thomas J. Peto, Rupam Tripura, Chan Davoeung, Chea Nguon, Sanann Nou, Chhouen Heng, Pich Kunthea, Bipin Adhikari, Renly Lim, Nicola James, Christopher Pell, Phaik Yeong Cheah

**Affiliations:** 1 Mahidol-Oxford Tropical Medicine Research Unit, Faculty of Tropical Medicine, Mahidol University, Bangkok, Thailand;; 2 Centre for Tropical Medicine and Global Health, Nuffield Department of Clinical Medicine, University of Oxford, Oxford, United Kingdom;; 3 Department of Infectious Diseases, Academic Medical Center, University of Amsterdam, Amsterdam, The Netherlands;; 4 Department of Provincial Health, Battambang, Cambodia;; 5 National Center for Parasitology, Entomology and Malaria Control, Phnom Penh, Cambodia;; 6 School of Pharmacy and Medical Sciences, University of South Australia, Adelaide, Australia;; 7 Faculty of Public Health and Policy, London School of Hygiene and Tropical Medicine, London, United Kingdom;; 8 Centre for Social Science and Global Health, University of Amsterdam, The Netherlands;; 9 Amsterdam Institute for Global Health and Development, Amsterdam, The Netherlands;; 10 The Ethox Centre, University of Oxford, Oxford, United Kingdom

## Abstract

Mass drug administration (MDA) to interrupt malaria transmission requires the participation of entire communities. As part of a clinical trial in western Cambodia, four villages received MDA in 2015–2016. Before approaching study communities, a collaboration was established with the local health authorities, village leaders, and village malaria workers. Formative research guided the development of engagement strategies. In each village, a team of volunteers was formed to explain MDA to their neighbors and provide support during implementation. Public mobilization events featuring drama and music were used to introduce MDA. Villages comprised groups with different levels of understanding and interests; therefore, multiple tailored engagement strategies were required. The main challenges were explaining malaria transmission, managing perceptions of drug side effects, and reaching mobile populations. It was important that local leaders took a central role in community engagement. Coverage during each round of MDA averaged 84%, which met the target for the trial.

The spread of drug-resistant parasites poses a serious threat to malaria control in Southeast Asia.^1^ In response, strategies to interrupt local malaria transmission, including mass drug administration (MDA), have been proposed.^2^ The success of this approach, currently under pilot across the region, depends upon high uptake in target communities.^3,4^ For past MDAs, this has been challenging^5^ because of misconceptions about drug regimens, inadequate explanations of the rationale for MDA, and limited awareness of disease risk and asymptomatic malaria.^6^ To overcome these challenges, a range of community engagement (CE) activities are undertaken alongside MDA.^7^ In the global health literature, CE has various definitions, for example, promoting ethical conduct of research, or “working collaboratively” with communities “to address issues affecting the well-being of those people.”^8,9^ In this article, we focus on CE as a range of activities with the primary aim of promoting MDA coverage.

Battambang Province, an area of unstable malaria transmission in western Cambodia, has seen a decline in clinical *Plasmodium falciparum* malaria over the past decade.^10^ Recently, *P. falciparum* parasites in the area have become resistant to artemisinins and partner drugs used in artemisinin combination therapy.^11–14^ Village malaria workers (VMWs), present in most villages, are trained to diagnose and treat clinical malaria. Asymptomatic malaria infections go untreated and contribute to transmission, and are associated with travel to forests and a history of clinical malaria.^15–19^ In neighboring Pailin Province, prevalence surveys (2013–2014) revealed a reservoir of asymptomatic malaria.^20^

As part of a clinical trial, two villages received MDA in 2015 and two in 2016. MDA consisted of three, monthly rounds of treatment with dihydroartemisinin–piperaquine (Figure 1). Participants were followed over one year to observe clinical malaria and cross-sectional surveys were conducted quarterly to determine the prevalence of asymptomatic malaria. This article describes the process of developing and implementing a CE strategy for MDA, outcomes, challenges, and lessons learned. To this end, we sought the views of study staff from all levels: policy-makers, scientists, and field-workers. The first step entailed the field team compiling a summary of the process of CE, describing the preparatory work, listing activities, and adding the lessons learned. Local staff (who are fluent in Khmer and familiar with the local population) made key contributions to this report.

**Figure 1.**
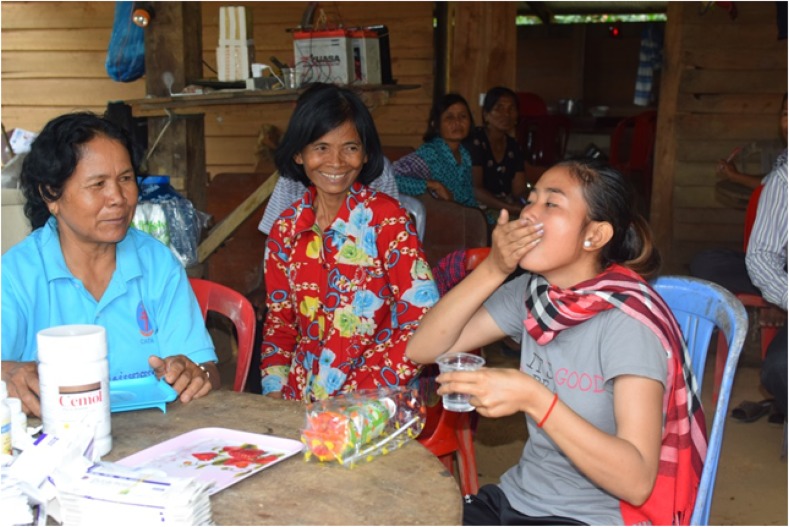
Participation in mass drug administration supervised by a village malaria worker and health center nurse with village leaders in the background. This figure appears in color at www.ajtmh.org.

Before approaching study communities, a series of meetings with provincial and local health authorities, village leaders, and VMWs enabled the study team to understand local political and social structures. Guided by local collaborators, the team spent several months conducting prevalence surveys to identify submicroscopic malaria and communities that would be suitable for MDA.^21^ Formative research was conducted in 12 selected villages to guide the CE strategy. Village leaders were interviewed using a semi-structured guide. The framework approach^22^ was used to identify themes to prioritize for engagement activities. This entailed developing a matrix of summarized responses according to the research themes for each respondent. Respondents recommended involving all political groups, VMWs, and government/private health staff in activities. Study-specific and general malaria education was recommended in light of villagers’ limited knowledge about malaria transmission. Preferred CE activities included video shows, quizzes with prizes, art and games, and musical concerts.

In 2015, local volunteers, VMWs, and village leaders joined the study team to conduct CE activities and MDA. Before MDA, research staff meet with different groups, such as mothers, school children, forest workers, and monks (Table 1). In every village, the major mobilization activity was a public concert where health information was presented. These were very popular and well-attended. To keep people informed and address issues as they arose, meetings and other activities continued during the three rounds of MDA. After the final round, the CE team continued to make weekly village visits to provide information about malaria and receive feedback. Before MDA in 2016, the engagement strategies were reviewed and lessons learned from 2015. An excess of detail about the study had reportedly confused people and staff worked with local leaders to simplify the messages. A drama-based event replaced the concert. This enabled local people to be involved as actors and communicate the scripted health education messages. In addition, health messages were given by the local health authorities known to the villagers instead of members of the research team.

**Table 1:** Engagement activities conducted before, during, and after mass antimalarial drug administration

Activity	Description	Aim
Leaders and influential people	Meetings were held with village leaders and influential people to introduce the project and plans for the year, and to obtain agreement for the whole village to work together.	Formal introduction of study, build relationships
Village volunteers	Volunteers were selected to cover a group of households within the village and were responsible for helping communicate with the community and also lead invitations and assist during survey days.	Capacity building, mobilizing participants, identifying problems
Involve village malaria workers	In groups and one-to-one, explain objectives again and to conduct participant selection and invitation process.	Ensure aims are understood, identify groups affected by malaria
Outreach activities with forest goers and migrants	Small meetings, visits to forest: teaching, health education, contacting them for surveys, encouraging participation in drug administration. Include migrants at risk of malaria who are professionals, such as soldiers and mine clearance teams.	Build knowledge among high-risk and hard-to-reach groups
Outreach to local opinion formers	Small group meetings with local political leaders, teachers, shopkeepers, private sector health-care providers, traditional healers.	Build relationships, avoid organized opposition to MDA
Outreach to monks	Visit pagoda, arrange day for monks to come for blessings, and talk on communities working together and the importance of health.	Collaborate with existing authorities, build relationships
Outreach to women/mothers	In small groups, teach, listen to, and address concerns about women or children taking medicine. Explain exclusion of pregnant and lactating women during MDA.	Build relationships, solicit views on MDA
Local school activities	Outdoor games, coloring in games, and prizes. Involving children in public performances ensures the parents will attend the event.	Fun activities, encourage participation, avoid fear
Post-MDA follow-up	Daily follow-up during drug administration to record and assist with any reports of real or perceived adverse events.	To identify any adverse events, ensure participant safety, avoid negative perceptions
Community concert (2015)	Band, quiz, prizes, invited speakers, household gift packs, and snacks (main mobilization event before MDA)	Provide information about MDA, build relationships trust
Community theater and art workshops (2016)	Video performance, drama workshops, singing competition, public drama performance (main mobilization event before MDA)	Provide information about MDA, build relationships trust
Incentives	Compensation was provided when individuals attended surveys or participated in MDA. For each event, participants received snacks and a reimbursement for their time of USD 2.5. In 2016 (after the clinical trial ended), no compensation was provided as MDA was conducted house-to-house and participation rates remained constant.	To reimburse time away from work and motivate continuous participation
Complimentary health care	A field clinic was conducted during each survey and round of MDA to provide free treatment to villagers.	Supporting healthcare in the community
Informed consent	Participants were gathered to explain malaria, MDA, and blood collection, through group presentations, and information was given using handouts, pictures, photos, and videos. Following this, written consent was obtained on an individual basis.	Clinical trial specific: provide information to support the consent process and obtain community approval
Monitoring and evaluation	Census of villages and major CE meetings: meeting with household heads and village leaders, review of population list, house-to-house follow-up, record keeping.	Collect feedback to adapt the CE strategy

CE = community engagement; MDA = mass drug administration.

All four invited villages agreed to participate in the study. Events such as meetings were well-attended and village malaria teams and volunteers were actively involved. For example, attendance for the drama performances ranged from 67% (350/522) to 86% (250/291). During each MDA round, coverage averaged 84%—exceeding the trial target.

There were several challenges when designing and implementing MDA. The rationale for MDA is difficult to convey, particularly explaining 1) asymptomatic malaria; 2) that asymptomatic infections contribute to transmission; 3) that to remove these infections people need to take drugs when they do not feel ill; and 4) that everyone in the village needs to participate for MDA to work. Within villages, education levels varied widely, and this had to be taken this into account when framing the rationale for MDA and key messages and tailoring engagement strategies (Table 2).

**Table 2:** Key experiences and lessons for mass antimalarial drug administration

Experience from MDA in the context of a clinical trial
Positives: well-resourced, novelty factor, compensation in 2015, small number of villages. Negatives: blood collection, trial procedures, such as consent forms, and study concepts, such as research and randomization are hard to convey
Messages
Messages need to be simple and consistent. The messengers need to first understand key concepts themselves. Smaller group meetings can be used to train locals who will implement MDA, followed by larger events to demonstrate popular and official support. Multiple engagement and education activities are recommended to reach all groups.
Explaining asymptomatic malaria and MDA
*“Malaria is caused by a tiny parasite that lives in people and feeds on blood. Some people may have malaria but do not feel sick. Mosquitos biting these people can spread malaria to other people. There are safe and effective medicines to cure malaria. If everybody in the village takes medicine at the same time then there will be nobody in the village who can infect other people. We call this MDA and we think it can stop people getting malaria. By joining MDA people can protect themselves and their families. After MDA people may still be at risk of malaria and so it is important to keep using bed nets and protection from mosquitos and still visit the village malaria worker if people think they may have malaria.”*
Explaining the study medicine and side effects
*“During MDA everyone will take a medicine called DHA-P for 3 days, then again next month and the month afterwards (three rounds in total). We know this medicine to be safe and effective and it has been used by village malaria workers for many years. All medicines can have side effects in some people but not all people get side effects. Most side effects from taking DHA-P are minor and do not last long. We do not expect that you will feel unwell after taking DHA-P, but if you do then inform a member of the MDA team and a nurse can come to help.”*
Lessons for implementation
Build on existing local resources, health services, and social structures and take sufficient time to prepare. Involve stakeholders in advance to build trust and understanding and overcome potential skepticism, fear, rumormongering, or political and social divisions. Providing MDA at a central location and house-to-house MDA are both acceptable. Public events, public censuses, public meetings, and public drug administration all mobilize the community and generate confidence about MDA. Engagement is important not only before MDA, but also during and after MDA to deal quickly and calmly with any real or perceived health issues that occur following treatment.

DHA-P = dihydroartemisinin–piperaquine; MDA = mass drug administration.

Before, during and after MDA, concerns from communities and staff emerged about perceived side effects of antimalarials. We gave directly observed treatment during MDA and then monitored participants actively for a week and passively for a month. Post-MDA, minor side effects such as dizziness, tiredness, and cold-like symptoms were reported in all sites. Local nurses and village volunteers visited participants to assist and reassure them as needed. Side effects were discussed directly in public meetings. Moreover, MDA was conducted as part of a clinical trial, and communities were generally unaware of medical research.

Another challenge was reaching migrants and forest workers, who are a group at higher risk of malaria infection but are more difficult to engage with as they often move in and out of the villages. Targeted meetings were held with these groups at times when they were not busy and MDA was provided when they returned from traveling if they were away during scheduled rounds of MDA. In one village, most people belonged to an ethnic minority, which meant the translation of messages into the local language. Their leader was bilingual and after being formally asked he joined the local MDA team to translate and successfully mobilized people from his community.

The experiences highlighted how understanding the concerns and attitudes of local communities and addressing them through various engagement activities are integral to the success of MDA. From our experience over two years, public events, such as concerts and drama, were important and ensured everybody received the same information at the same time. The events had to be entertaining to attract large audiences, which also built relationships and generated trust between the research team and communities.

As challenges often arose over time, a process of ongoing contact through meetings and regular visits with families was important. This highlights that multiple complementary engagement strategies were required. Participating in the MDA depended also on people’s trust, and local leaders were able to advise on the language and methods to communicate the rationale as well as taking a leading role in community mobilization.

MDA that takes place under non-trial “real-life” conditions will face additional challenges because it will need to be organized largely by communities themselves, and probably with fewer resources. At scale, effective community mobilization and local ownership will be essential if MDA is to be successful and sustainable over several years. CE will need to build upon existing local social structures: community leaders, government health staff, and village health workers, and other representatives of the local community, such as women’s leaders and forest workers. Without a large outside team to organize MDAs, local villages will need to create their own teams to mobilize their communities and educate them about malaria transmission and the rationale for MDA, including visits and follow-up of every household to ensure people participate during each round. Local capacity will need to be developed to enable villagers to lead MDAs. Well-designed education materials and support from local authorities and health workers will be needed, including training of village MDA teams to increase health literacy, support the organization of MDA, and keep accurate records. Motivated local MDA teams, with appropriate support, would be well placed to understand the concerns of their own communities and plan how to implement MDA in a locally adapted way to reach all parts of their community.

In Southeast Asia, mass antimalarial administration for malaria elimination may be targeted at villages with proven reservoirs of asymptomatic malaria rather than over wider areas, requiring the engagement of individual communities. In Cambodia, a pilot MDA achieved good coverage and this may be attributable to close collaboration with national and local authorities and a community-directed engagement strategy.

## References

[1] Ashley EA 2014 Spread of artemisinin resistance in *Plasmodium falciparum* malaria. N Engl J Med 371: 411–423. 25075834 10.1056/NEJMoa1314981 PMC4143591

[2] Dondorp AM Smithuis FM Woodrow C Seidlein LV, 2017 How to contain artemisinin- and multidrug-resistant *falciparum* malaria. Trends Parasitol 33: 353–363. 28187990 10.1016/j.pt.2017.01.004

[3] World Health Organization, 2014 *Accelerating Malaria Elimination in the Greater Mekong Subregion* Available at: http://www.who.int/malaria/areas/greater_mekong/overview/en/. Accessed July 13, 2017.

[4] World Health Organization, 2015 *Strategy for Malaria Elimination in the Greater Mekong Subregion (2015–2030)* Available at: http://iris.wpro.who.int/bitstream/handle/10665.1/10945/9789290617181_eng.pdf?sequence=1. Accessed July 14, 2017.

[5] Sturrock HJ Hsiang MS Cohen JM Smith DL Greenhouse B Bousema T Gosling RD, 2013 Targeting asymptomatic malaria infections: active surveillance in control and elimination. PLoS Med 10: e1001467. 23853551 10.1371/journal.pmed.1001467 PMC3708701

[6] Cantey PT Rout J Rao G Williamson J Fox LM, 2010 Increasing compliance with mass drug administration programs for lymphatic filariasis in India through education and lymphedema management programs. PLoS Negl Trop Dis 4: e728. 20628595 10.1371/journal.pntd.0000728 PMC2900179

[7] Adhikari B James N Newby G von Seidlein L White NJ Day NP Dondorp AM Pell C Cheah PY, 2016 Community engagement and population coverage in mass anti-malarial administrations: a systematic literature review. Malar J 15: 523. 27806717 10.1186/s12936-016-1593-y PMC5093999

[8] Clinical and Translational Sciene Awards (CTSA), 2011 *Principles of Community Engagement*. NIH Publication No. 11-7782; second edition.

[9] Tindana PO Singh JA Tracy CS Upshur RE Daar AS Singer PA Frohlich J Lavery JV, 2007 Grand challenges in global health: community engagement in research in developing countries. PLoS Med 4: e273. 17850178 10.1371/journal.pmed.0040273 PMC1989740

[10] Maude RJ 2014 Spatial and temporal epidemiology of clinical malaria in Cambodia 2004–2013. Malar J 13: 385. 25266007 10.1186/1475-2875-13-385 PMC4531392

[11] Phyo AP 2012 Emergence of artemisinin-resistant malaria on the western border of Thailand: a longitudinal study. Lancet 379: 1960–1966. 22484134 10.1016/S0140-6736(12)60484-X PMC3525980

[12] WHO, 2010 *Global Report on Antimalarial Drug Efficacy and Drug Resistance: 2000–2010*. Geneva, Switzerland: World Health Organization.

[13] Dondorp AM 2009 Artemisinin resistance in *Plasmodium falciparum* malaria. N Engl J Med 361: 455–467. 19641202 10.1056/NEJMoa0808859 PMC3495232

[14] Noedl H Se Y Schaecher K Smith BL Socheat D Fukuda MM; Artemisinin Resistance in Cambodia 1 Study Consortium, 2008 Evidence of artemisinin-resistant malaria in western Cambodia. N Engl J Med 359: 2619–2620. 19064625 10.1056/NEJMc0805011

[15] Durnez L Mao S Denis L Roelants P Sochantha T Coosemans M, 2013 Outdoor malaria transmission in forested villages of Cambodia. Malar J 12: 329. 24044424 10.1186/1475-2875-12-329 PMC3848552

[16] Peto TJ 2016 History of malaria treatment as a predictor of subsequent subclinical parasitaemia: a cross-sectional survey and malaria case records from three villages in Pailin, western Cambodia. Malar J 15: 240. 27118311 10.1186/s12936-016-1284-8 PMC4845326

[17] Peto TJ 2016 Association between subclinical malaria infection and inflammatory host response in a pre-elimination setting. PLoS One 11: e0158656. 27386859 10.1371/journal.pone.0158656 PMC4936742

[18] Imwong M 2015 The epidemiology of subclinical malaria infections in South-East Asia: findings from cross-sectional surveys in Thailand–Myanmar border areas, Cambodia, and Vietnam. Malar J 14: 381. 26424000 10.1186/s12936-015-0906-x PMC4590703

[19] Imwong M 2016 Numerical distributions of parasite densities during asymptomatic malaria. J Infect Dis 213: 1322–1329. 26681777 10.1093/infdis/jiv596 PMC4799672

[20] Tripura R 2016 Persistent *Plasmodium falciparum* and *Plasmodium vivax* infections in a western Cambodian population: implications for prevention, treatment and elimination strategies. Malar J 15: 1–12. 27013512 10.1186/s12936-016-1224-7 PMC4806483

[21] Tripura R 2017 Submicroscopic *Plasmodium* prevalence in relation to malaria incidence in 20 villages in western Cambodia. Malar J 16: 56. 28143518 10.1186/s12936-017-1703-5 PMC5282880

[22] Gale NK Heath G Cameron E Rashid S Redwood S, 2013 Using the framework method for the analysis of qualitative data in multi-disciplinary health research. BMC Med Res Methodol 13: 117. 24047204 10.1186/1471-2288-13-117 PMC3848812

